# Soluble Receptor for Advanced Glycation End Products (sRAGE) Level and Its Prognostic Significance in Children with Acute Lymphoblastic Leukemia

**DOI:** 10.3390/children11020176

**Published:** 2024-01-31

**Authors:** Busra Ozkan, Yasemin Altuner Torun, Cigdem Karakukcu, Binnaz Celik

**Affiliations:** 1 Department of Pediatrics, Beylikduzu Public Hospital, Istanbul 34500, Turkey; busra.ozkan13@saglik.gov.tr; 2 The Faculty of Medicine, Department of Child Hematology and Oncology, Istinye University, Istanbul 34510, Turkey; yasemin.altuner@istinye.edu.tr; 3 The Faculty of Medicine, Department of Biochemistry, Erciyes University, Kayseri 38039, Turkey; ckarakukcu@erciyes.edu.tr; 4 Department of Pediatrics, Kayseri City Education and Research Hospital, Kayseri 38080, Turkey

**Keywords:** ALL, sRAGE, diagnosis, children

## Abstract

Acute lymphoblastic leukemias are the most common malignancies in childhood. Although its etiology is still unclear, it is thought that disorders in oxidative stress metabolism may contribute to leukemogenesis. Advanced glycation end products (AGEs) are formed as a result of the non-enzymatic binding of sugars to biomolecules. Oxidation reactions are triggered through AGE–Receptor (RAGE) interaction, resulting in the formation of reactive oxygen species. These can play crucial roles in cancer pathogenesis and leukemogenesis. It is thought that sRAGE (soluble RAGE) is the end product of glycation and circulates freely in the circulation by binding to RAGE ligands. We investigate novel leukemia biomarkers and focus on soluble RAGE (sRAGE) for acute lymphoblastic leukemia (ALL) diagnosis and prognosis. Thirty children (1–17 years) diagnosed with ALL were included in the study. Patients were divided into standard, medium, and high risk groups according to the Berlin–Frankfurt–Münster (BFM) treatment protocol. Patients were evaluated twice; at the time of diagnosis and at the sixth month of remission. sRAGE and blood parameters were compared with healthy controls (*n* = 30, 1–17 years). The sRAGE levels in ALL patients at diagnosis (138.7 ± 177.3 pg/mL) were found to be significantly higher than they were during the sixth month of remission (17.6 ± 21.1 pg/mL) and in healthy controls (22.2 ± 23.7 pg/mL). The cut-off value of the sRAGE level for the diagnosis of ALL was found to be 45 pg/mL in ROC analysis (sensitivity: 73.3%, specificity: 86.7%, AUC: 0.681). At the same time, the sRAGE level was found to be significantly higher in T-ALL patients (490.9 ± 236.9 pg/mL) than in B-ALL patients (84.5 ± 82.7 pg/mL). No significant difference was found in terms of the sRAGE level between standard (45.8± 33.1 pg/mL), medium (212 ± 222.1 pg/mL), and high (143.9 ± 111.5 pg/mL) risk group ALL patients classified according to the BFM protocol. Despite the fact that this was a small, single-center study, our findings highlight the potential use of sRAGE as a biomarker for diagnosing ALL and assessing response to treatment.

## 1. Introduction

Leukemias arise from hematopoietic stem cells. Acute lymphoblastic leukemia (ALL) is a malignancy that occurs when lymphoid cells undergo arrest at a certain stage of hematopoiesis, leading to the uncontrolled proliferation of clonal cells that have not completed their differentiation and which subsequently infiltrate the bone marrow [1,2]. Acute lymphoblastic leukemia is the most common childhood malignancy and constitutes approximately more than 50% of all childhood hematopoietic malignancies [3,4]. In contrast, it is a much rarer hematological malignancy in adults [4]. The disease may arise from different lymphocyte subsets that cause B-cell (85%) and T-cell (15%) leukemias, sometimes causing mixed leukemias [5]. During the course of the disease, normal bone marrow function deteriorates, and anemia, thrombocytopenia, and neutropenia develop. Patients may experience symptoms such as weakness, bone pain, pallor, bleeding, and infection [6]. The goal of ALL treatment is to quickly destroy as many leukemia cells as possible and achieve normal complete blood count values. Therefore, treatment begins with remission induction through the use of combined chemotherapeutics. This treatment is followed by consolidation and maintenance therapy to destroy the few remaining leukemic cells in the blood or bone marrow [7].

Although the exact etiology of leukemia is unknown, genetic and environmental factors play crucial roles [8]. Oxidative damage causing infection, inflammation, and mutation can also transform leukemic cells. Oxidative stress can be defined as the imbalance between the production of reactive oxygen species and their neutralization [8,9]. Oxidative stress metabolism disorders are known to be a common feature of mutant tumor cells. It is also thought that the products formed as a result of oxidation reactions can be used in cancer treatment by inducing apoptosis [10]. However, information concerning pretreatment oxidative damage and the antioxidant system in newly diagnosed ALL patients is limited and contradictory [6]. Leukemic cells may produce free oxygen radicals, and there is a relationship between free oxygen radical activity and leukemogenesis [8]. There are other mechanisms that can cause carcinogenesis, and one of them is glycative stress, which is less studied than oxidative stress [11].

The non-enzymatic addition of sugars to nucleic acids, proteins, and lipids results in the formation of advanced glycation end products (AGEs) through oxidative and/or non-oxidative reactions [12]. The interaction of AGEs with the AGE receptor (RAGE) may result in the generation of reactive oxygen species. Recent research has underscored the importance of AGEs and their receptor RAGE as crucial contributors to the etiopathogenesis of chronic inflammation and multiple diseases [13,14,15]. Inflammation is thought to be a risk factor for the formation of many cancers, including leukemic blasts [12]. Through its involvement in critical cellular functions such as the immune inflammatory response, apoptosis, and endothelial dysfunction, RAGE plays a pivotal role in triggering these diverse conditions [16].

The RAGE protein is expressed as a soluble isoform (sRAGE) or a full-length RAGE isoform (fl-RAGE; mRAGE), which are bound to cell membranes. It has been reported that sRAGE, the soluble receptor of the final metabolite product of glycation, is freely found in tissue and circulation and acts as a decoy receptor that captures and binds RAGE ligands in the extracellular space. sRAGE is a variant of RAGE that carries all extracellular domains but does not contain the intracellular and transmembrane portion of full RAGE. Therefore, it acts as an endogenous competitive fl-RAGE inhibitor. It has also been shown to inhibit RAGE-induced cellular signaling, ultimately leading to tissue damage [17,18]. Additionally, sRAGE blood levels increase in relation to pathological pro-inflammatory processes and can be used as a biomarker of disease risk and negative outcomes [19].

AGE and sRAGE levels have been studied in various types of cancer and chronic diseases, but clear information is not yet available. Although the importance of oxidative damage and chronic inflammation in leukemogenesis is known, no study examining the sRAGE level in ALL has been found in the literature. In this study, the sRAGE levels of patients with acute lymphoblastic leukemia at the time of diagnosis and at the sixth month of maintenance treatment (remission period) were compared with the levels observed in healthy controls. Thus, this study aimed to determine the importance of the sRAGE level in the diagnosis and prognosis of ALL. It also aimed to compare the sRAGE level between T-cell and B-cell ALL patients.

## 2. Materials and Methods

Thirty patients (aged 1–17 years) who were newly diagnosed with ALL in the Hematology Oncology Clinic of Kayseri Education and Research Hospital were included in the study. The control group consisted of healthy children in the same age range who presented at outpatient clinics for any reason and without underlying chronic disease or active infection.

Bone marrow aspiration was performed in patients suspected of having leukemia as a result of their medical history, physical examination, and routine laboratory tests. A diagnosis of leukemia was made with the observation of blastic cells in the bone marrow examination, and classified using immunophenotyping via flow cytometry. As a result of the classification, patients diagnosed with ALL were included in this study. According to the Berlin–Frankfurt–Münster (BFM) protocol [20], ALL patients were divided into three different risk groups to determine the treatment strategy.

High risk group (HRG): Prednisolone poor response on day 8, no complete remission on day 33, presence of t (4;11) and/or t (9;22).

Medium risk group (MRG): Initial white blood cell count (WBC) > 20.000/mm^3^ and/or age at diagnosis < 1 years or >6 years, prednisolone good response on day 8, complete remission on day 33, T-cell ALL.

Standard risk group (SRG): Initial WBC < 20.000/mm^3^, age at diagnosis between 1 and 6 years, good prednisolone response on day 8, complete remission on day 33, no T-cell ALL.

Following diagnosis and risk stratification, appropriate treatment was started. The children were first given remission induction therapy for 4 weeks. As remission induction treatment, three- or four-drug (vincristine, dexamethasone/prednisone, L-asparaginase, and/or anthracycline) chemotherapy and intrathecal treatment were started for the children, taking into account their risk classification. Patients who were found to have complete remission in the bone marrow examination performed on the 33rd day of this treatment were re-evaluated as the remission group in the sixth month of maintenance treatment. Laboratory parameters evaluated at the time of diagnosis were taken again.

The demographic data of the patients and control individuals were recorded. Blood samples from the patient group were taken at the time of diagnosis, before any chemotherapy or anti-inflammatory drugs were administered. Among the patients admitted at the time of diagnosis or during the remission period, those with active infection and underlying chronic diseases were excluded from this study. Blood was taken from all three groups to determine the complete blood count, routine biochemical parameters, erythrocyte sedimentation rate (ESR), and sRAGE levels.

Approval was obtained from the clinical research ethics committee on 30 April 2020, and the study was numbered 36. A consent form outlining the study was signed by the parents of all patients and healthy children participating in the study after they were provided with information.

### 2.1. Measurement of Complete Blood Count and ESR

The complete blood count was analyzed through cytometry using a complete blood count auto-analyzer (Sysmex XN-9000, Sysmex Corporation, Kobe, Japan), and ESR was analyzed using a vision automatic sedimentation analyzer with the Westergren method.

### 2.2. Measurement of Serum Biochemical Parameters

The biochemical parameters (blood urea nitrogen (BUN), uric acid, lactate dehydrogenase (LDH), creatinine, albumin, aspartate aminotransferase (AST), alanine aminotransferase (ALT), and alkaline phosphatase (ALP)) were analyzed spectrophotometrically using Roche Cobas 8000 brand modular auto-analyzers (Roche Diagnostics, Mannheim, Germany) with kits belonging to the manufacturer.

### 2.3. Serum sRAGE Level Measurement

To measure of the serum sRAGE levels of the patients (at the time of diagnosis and during the sixth month remission) and control groups, 2 mL blood samples were placed into gel biochemistry tubes and kept at +4 °C for 15 min. The blood samples were centrifuged at 3500 rpm for 15 min. After the obtained serum samples were separated into Eppendorf tubes, they were stored at −80 °C in a deep freezer until analysis. The serum sRAGE level was analyzed in our hospital’s biochemistry laboratory using the enzyme-linked immunosorbent assay (ELISA) method and a commercial kit according to the manufacturer’s instructions (Elabscience Biotechnology Inc., Houston, TX, USA). The serum sRAGE level was expressed as pg/mL. The intra-/inter-assay coefficients of variation were <10%. The concentrations of the samples were calculated using the calibration curve obtained from known levels of analysis standards.

### 2.4. Statistical Analysis

All analyses were conducted using the SPSS 26 program. The mean ± standard deviation, median (lowest–highest), frequency, and ratio were used for the descriptive statistics of the data. The Kolmogorov–Smirnov test was used to evaluate the distribution of the variables. To compare the difference between groups, the Mann–Whitney U, Kruskal–Wallis test, and Wilcoxon test were used for non-parametric data, and an independent sample *t*-test and paired sample *t*-test were used for the parametric data. The chi-square test was used for qualitative independent data analysis. ROC (receiver operating characteristic) analysis was used to evaluate whether the sRAGE value could be used in the diagnosis of ALL and to determine a predictive value. For all comparisons, a value of *p* < 0.05 was considered statistically significant.

## 3. Results

**Figure 1:**
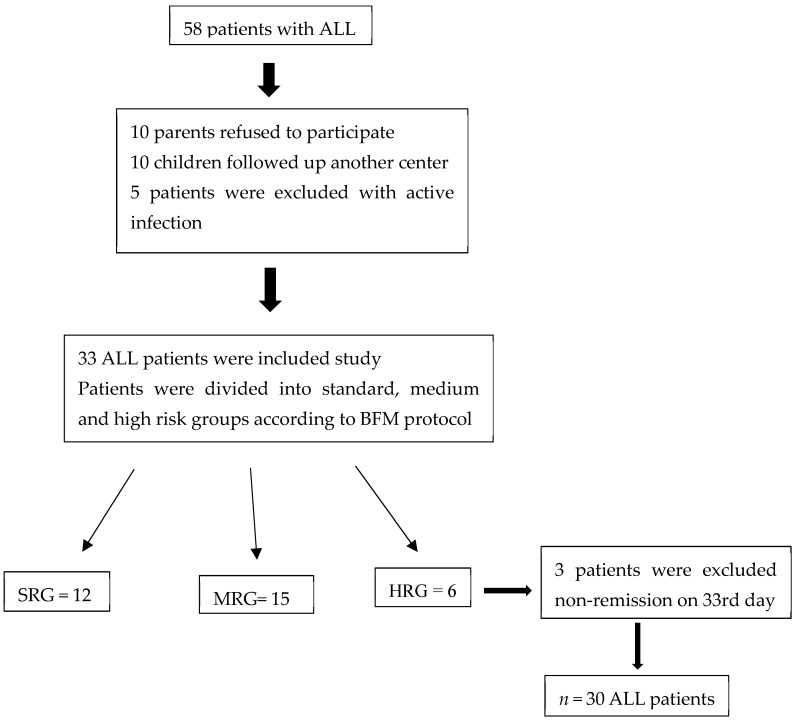
Flow chart of ALL patients.

During the time of the study, 58 newly diagnosed ALL patients were admitted to our hematology–oncology clinic. In total, 10 parents of these patients refused to participate in the study, and 10 patients did not attend the follow-up because they were still being followed-up at another center during the remission period. A total of five patients were excluded from the study due to active infection at the time of diagnosis. A total of 33 patients and a control group of 30 individuals of similar ages (1–17 years) were included in the study. None of our ALL patients had t (4;11) or t (9;22) translocation. According to BFM risk classification, 12 patients were in the standard, 15 patients were in the medium risk group, and 6 patients were in the high risk group on the eighth day of remission induction treatment. Three of the six patients in the high risk group were excluded from the study because no remission was observed in the bone marrow control on the 33rd day. Thus, 30 ALL patients were compared with 30 healthy controls at the time of diagnosis and at the sixth month of remission (Figure 1).

The patient group (30) and control group consisted of 17 boys (56.7%) and 13 girls (43.3%). The age of the patients ranged from 1 to 17 years; the mean age was 7 ± 3.7 years, and that of the control group was 6 ± 4.2 years. The mean age of the male patients was 6.18 ± 4.9 years, while that of the female patients was 5.85 ± 2.9 years. There was no significant difference in terms of age or sex between the patient and control groups.

The complete blood count parameters of the patient and control group are summarized in Table 1. Neutrophil (1.3 ± 1.5 × 10^3^/μL) counts were significantly lower in the patient group at diagnosis than in the control group (3.4 ± 1.4 × 10^3^/μL). However, at the time of diagnosis, the lymphocyte counts in the patient group were higher (19.5 ± 40.5 × 10^3^/μL) than in the control group (3.2 ± 1.5 × 10^3^/μL), but this increase was not statistically significant.

Table 2 shows a comparison between the patients at diagnosis, patients during the sixth month of remission, and the control groups in terms of the biochemical parameters and erythrocyte sedimentation rate. However, the erythrocyte sedimentation rate was significantly greater in the patient group than in the control group.

**Figure 2:**
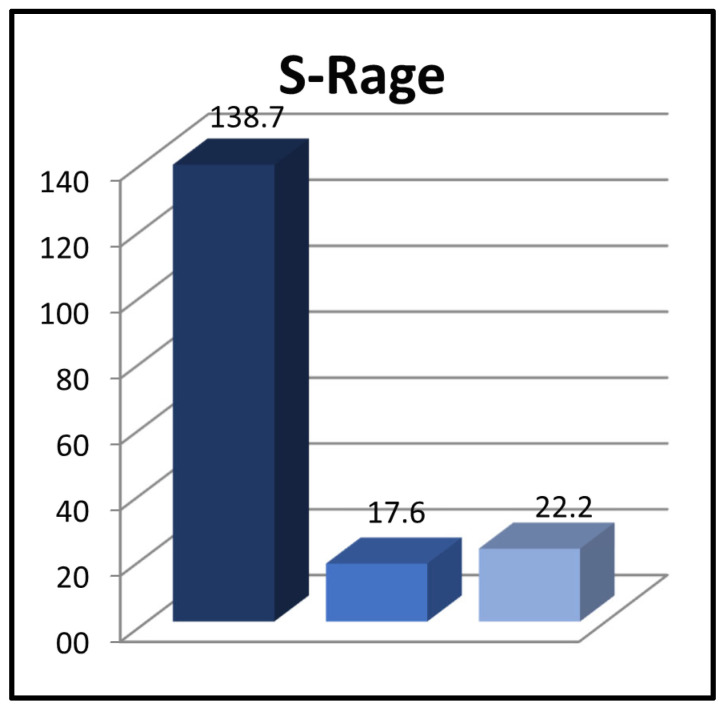
Comparison of sRAGE between the groups.

When comparing the levels of sRAGE among patients at diagnosis (138.7 ± 177.3 pg/mL), patients during the sixth month of remission (17.6 ± 21.1 pg/mL), and the control group (22.2 ± 23.7 pg/mL), it was observed that the levels were significantly higher in the patient group at the time of diagnosis than in both of the other groups (*p* = 0.000 and *p* = 0.000, respectively) (Figure 2).

**Figure 3:**
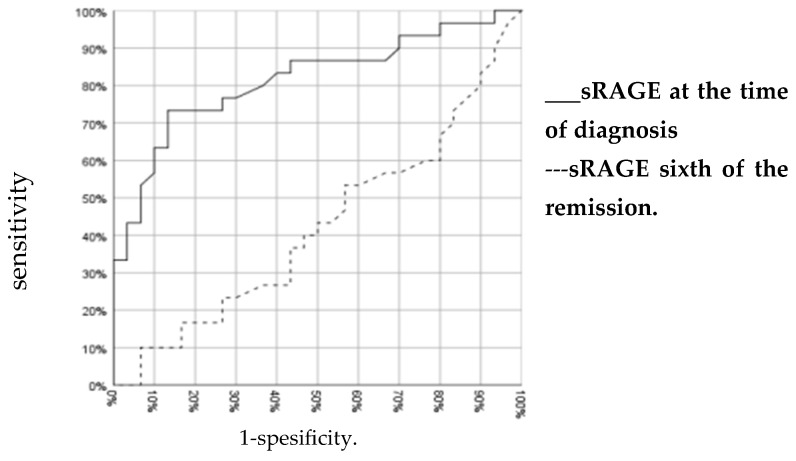
ROC curve for sRAGE.

The sRAGE cut-off value was evaluated with the ROC curve to separate patients diagnosed with ALL as a result of clinical and laboratory findings from the healthy control group. To diagnose ALL, sRAGE 45 pg/mL was determined as the cut-off value with 73.3% sensitivity and 86.7% specificity (area under the curve = 0.816) (Figure 3).

In B-ALL patients (*n* = 26), the mean sRAGE concentration was 84.5 ± 82.7 pg/mL at the time of diagnosis; in T-ALL patients (*n* = 4), the mean was 490.9 ± 236.9 pg/mL. The sRAGE level was significantly higher in T-ALL patients (*p* = 0.003) than in B-ALL patients (Table 3).

When the three ALL risk groups were compared in terms of sRAGE levels at diagnosis and during the sixth month of remission, no significant difference was found between the groups. The lack of significant difference in triple comparisons was attributed to the limited number of patients. When each group was compared in pairs, a significant difference was found between the sRAGE levels of the standard and medium risk groups at the time of diagnosis and during the sixth month of remission period (Table 4).

## 4. Discussion

The interaction of AGEs with their receptor (RAGE) causes oxidative damage and chronic inflammation. The importance of oxidative damage and chronic inflammation in carcinogenesis and leukemogenesis has been demonstrated in many studies. Circulating sRAGE is thought to play an important role in anti-inflammatory stress and disease protection by blocking AGE–RAGE interaction. On the other hand, there is another view that high sRAGE levels may exacerbate the pathological processes that occur as a result of excessive RAGE stimulation on the cell surface [18].

Our study showed that the sRAGE level was higher in patients with acute lymphoblastic leukemia at the time of diagnosis than in patients at the sixth month of remission and in the control group. We also found that the sRAGE level was significantly higher in T-cell ALL patients than in B-cell ALL patients. However, when evaluated according to ALL risk groups, we did not find significant differences in sRAGE levels.

In the literature, it has been reported that in the complete blood count of ALL patients with normocytic normochromic anemia, frequently increased or rarely decreased white blood cell count, neutropenia, and thrombocytopenia have been observed. This is due to the infiltration of clonal proliferating leukemic cells and a decrease in other cell lineages due to impaired normal bone marrow function [21]. In a study involving 203 ALL patients [22], 27.1% of the patients presented with leukocytosis and bicytopenia, 26% exhibited leukopenia and bicytopenia, and 17% displayed a normal leukocyte count along with bicytopenia. In accordance with the findings of the present study, the absolute neutrophil, eosinophil, and platelet counts were lower in our patient group at diagnosis than in the control group.

There are no studies in the literature evaluating sRAGE levels in patients with acute lymphoblastic leukemia. However, the role of the AGE–RAGE signaling pathway and sRAGE has been evaluated in various chronic diseases and cancers. In RAGE–ligand interactions, signal transmission pathways are activated, mediating increased intracellular oxidative stress and pro-inflammatory cytokine production, cellular functions such as proliferation, apoptosis, angiogenesis, migration, and fibrosis, as well as the malignant transformation of epithelial cells due to chronic inflammation [23,24]. This chronic inflammation can cause carcinogenesis and leukemic transformation of hematopoietic cells [13]. At this point, sRAGE prevents the proliferation and invasion of tumor cells by binding ligands as decoy receptors outside the cell, preventing the interaction of ligands with membrane-bound RAGE, as well as the negative effects of AGE–RAGE signaling [25]. The high level of sRAGE in our patients at the time of diagnosis suggested that it may have increased to compensate for the inhibition of AGE–RAGE signaling and leukemogenesis.

Although there are no studies on glycative stress in patients with acute lymphoblastic leukemia, oxidative stress plays an important role. In a study evaluating oxidative stress in children diagnosed with ALL [9], it was determined that reactive oxygen products and antioxidant enzymes play important roles in both leukemia development and leukemic relapse. In our study, sRAGE, an indicator of glycative stress, was evaluated; sRAGE levels were found to be significantly higher in patients diagnosed with ALL at the time of diagnosis.

sRAGE levels have been evaluated in various cancer types and different results have been obtained. sRAGE levels were previously found to be significantly higher in breast cancer patients compared to healthy controls [26]. Although sRAGE levels were found to be high in patients with pancreatic cancer, this was not found to be statistically significant [27]. The increase in sRAGE in these cancers has been explained as a compensation mechanism for the inhibition of inflammation and carcinogenesis that develops as a result of the AGE–RAGE interaction. There is no clear situation regarding the serum sRAGE level in lung cancer patients. Although there are studies stating that it decreases [28], there are also studies indicating that there is no difference in lung cancer patients compared to other inflammatory conditions [29].

Serum sRAGE levels were found to be high in patients with endometrial cancer and were found to be positively correlated with high tumor histological grade [30]. However, various clinical studies have shown a strong relationship between RAGE expression and the aggressiveness of various types of cancer [31]. From this perspective, the high sRAGE levels in the period of the highest tumoral load at the time of diagnosis and the decreased sRAGE levels in remission were similar in our patients. Moreover, sRAGE levels were found to be higher in T-ALL patients, which is associated with poor prognosis, than in B-ALL patients. However, we did not find any significant difference in sRAGE level between ALL risk groups. We think that this is due to the small number of patients in the risk groups.

In light of all these studies, it may seem more correct to state that sRAGE plays a role as a decoy receptor to prevent chronic inflammation, oxidative damage, and carcinogenesis that develop as a result of AGE–RAGE interaction which is increased in these cases. At the same time, the similarity between the sRAGE level in the remission period and the control group also suggests that the sRAGE level is highest in the proliferative period of the disease and decreases similar to LDH as the cellular turnover returns to normal. However, more comprehensive studies on different diseases and different age groups are needed to prove these suggestions.

## 5. Conclusions

Our study, in which the sRAGE level was evaluated in childhood ALL patients for the first time, is important as it shows that glycative stress, as well as oxidative stress, may be important in leukemogenesis. However, the fact that the study was conducted in a single center and with a low number of patients constitutes the limitations of our study. We believe that multicenter studies involving a larger patient cohort are necessary to elucidate the significance of sRAGE and the RAGE pathway in the development of ALL. These studies may be helpful in determining the use of sRAGE as a potential biomarker for diagnosis and may act as a guide to determine the effectiveness of treatment.

## Figures and Tables

**Table 1:** Comparison of complete blood count parameters between the groups.

Parameters (10^3^/μL)	Patient at Diagnosis	Patient during the Sixth Month of Remission	Control Group	*p* *
WBC	33.5 ± 60.5	5.6 ± 2.5	7.4 ± 2.5	>0.05
	*p* = 0.004			
Neutrophil	1.3 ± 1.5	3.3 ± 2.1	3.4 ± 1.4	0.000
	*p* = 0.000			
Lymphocytes	19.5 ± 40.5	1.7 ± 1.0	3.2 ± 1.5	>0.05
	*p* = 0.000			
Eosinophils	0.0627 ± 0.0871	0.1225 ± 0.1026	0.1657 ± 0.1617	0.000
	*p* = 0.036			
Platelets	89.3 ± 68.5	240.3 ± 60.9	329.8 ± 106.6	0.000
	*p* = 0.000			
Hemoglobin (gr/dL)	7.7 ± 2.4	13.8 ± 1.0	13.5 ± 1.3	0.000
	*p* = 0.000			

* Between patients in the diagnosis and control groups.

**Table 2:** Comparison of biochemical parameters between groups.

Parameters	Patient at Diagnosis	Patient during the Sixth Month of Remission	Control Group	*p* *
Albumin (g/L)	3.77 ± 0.57	4.58 ± 0.30	4.29 ± 0.44	0.000
	*p* = 0.000			
LDH (U/L)	1023.4 ± 1386.5	242.1 ± 8.4	233.1 ± 39.5	0.000
	*p* = 0.003			
AST (U/L)	61.0 ± 78.2	27.5 ± 8.4	25.0 ± 12.9	0.001
	*p* = 0.003			
ALT (U/L)	50.2 ± 91.0	33.6 ± 28.1	14.2 ± 9.8	0.000
	***p*** > 0.05			
ALP (U/L)	157.8 ± 93.2	267.4 ± 279.9	189.5 ± 95.7	0.095
	*p* = 0.002			
Kreatinin (mg/dL)	0.42 ± 0.21	0.39 ± 0.14	0.50 ± 0.14	0.006
	***p*** > 0.05			
Ürik Asit (mg/dL)	5.80 ± 3.73	3.51 ± 1.35	3.36 ± 1.0	0.002
	*p* = 0.000			
BUN (mg/dL)	12.29 ± 3.61	10.53 ± 2.77	10.13 ± 1.70	0.016
	***p*** > 0.05			
ESR (mm/h)	25.27 ± 16.43	6.36 ± 5.44	5.93 ± 3.86	
	*p* = 0.000			0.000

* Between patients at diagnosis and control groups.

**Table 3:** Comparison of sRAGE levels between the B-ALL and T-ALL patients and control group.

	B-ALL	T-ALL	Control Group	*p*
sRAGE at the time of diagnosis (pg/mL)	84.5 ± 82.7 *	490.9 ± 236.9 **	22.2 ± 23.7	* 0.001 ** 0.000
	0.003			
sRAGE during the sixth month of remission (pg/mL)	11.9 ± 12.0	55.0 ± 30.9		
	0.013			

* Between control group and B-ALL; ** Between control group and T-ALL.

**Table 4:** Comparison of sRAGE between ALL risk groups.

	SRG	MRG	HRG	*p*
sRAGE at diagnosis (pg/mL)	45.8 ± 33.1	212 ± 222.1	143.9 ± 111.5	>0.05
	0.025			
sRAGE during the sixth month of remission (pg/mL)	7.8 ± 8.4	26.6 ± 25.9	11.8 ± 11	>0.05
	0.017			

## Data Availability

The data presented in this study are available on request from the corresponding author. The data are not publicly available due to ethical restrictions.
